# Valorization of sorghum ash with digestate and biopreparations in the development biomass of plants in a closed production system of energy

**DOI:** 10.1038/s41598-023-45733-9

**Published:** 2023-10-30

**Authors:** Zdzisława Romanowska-Duda, Regina Janas, Mieczysław Grzesik, Bert van Duijn

**Affiliations:** 1 University of Lodz, Faculty of Biology and Environmental Protection, Banacha 12/16, 90-237 Lodz, Poland; 2 Cultivar Testing, Nursery and Gene Bank Resources Department, The National Institute of Horticultural Research, Konstytucji 3 Maja 1/3, 96-100 Skierniewice, Poland; 3 Plant Biodynamics Laboratory, Institute of Biology Leiden, Sylviusweg 72, 2333 BE Leiden, The Netherlands; 4 Fytagoras, Sylviusweg 72, 2333 BE Leiden, The Netherlands

**Keywords:** Plant sciences, Environmental sciences

## Abstract

Replacing chemical fertilizers with non-toxic waste that meet all fertilizing purposes, including ash from plant biomass and their management is becoming the important goal of sustainable agriculture concerning energy plants production in a closed system. This study aims to explore a novel strategy for utilizing natural sorghum ash together with digestate and ecological compounds, to replace synthetic fertilizers, for the energy plant development improvement and thus reduction of the environment pollution. Sorghum, as an energy plant, cultivated in low quality sandy and podzolic soils, in Central and North Poland climate, was fertilized with different doses of YaraMila Complex, a synthetic fertilizer (0, 150, 300 kg ha^−1^ Each dose was supplemented with different amounts of sorghum ash (0.5, 1, 2 and 4 t ha^−1^), used alone or with addition of APOL-HUMUS (soil improver; 10 L ha^−1^), biogas plant digestate (30 m^3^ ha^−1^) and Stymjod (nano-organic leaf fertilizer; 5 L ha^−1^). Added to each YaraMila Complex dose, the applied ash amounts (optimally 2–4 t ha^−1^), increased growth of plants, crop biomass, index of chlorophyll content, net photosynthesis, transpiration, stomatal conductance, content of intercellular CO_2_, activity of acid and alkaline phosphatase, RNase and dehydrogenase and energy properties. Sorghum ash used with the lesser YaraMila Complex doses of 0 or 150 kg ha^−1^ caused the enhanced growth of plants more than the doubled YaraMila Complex amounts applied alone (150 or 300 kg ha^−1^, correspondingly). Additionally, applied biogas plant digestate, APOL-HUMUS and Stymjod further increased the plant growth. This indicates that the application of natural sorghum ash accelerates energy plant development, can reduce by half the recommended synthetic fertilizer doses on poor and marginal soil and enables the cultivation of sorghum in a closed production cycle.

## Introduction

The management of non-toxic natural waste that meet all fertilizing requirements and can replace the synthetic fertilizers is becoming one of the most important goals in current agriculture. A relatively small number of studies indicate that one of such bio-fertilizers may be the natural ash from annual plants, including cereals, provided that it contains macro- and microelements necessary for plant development and is free of heavy metals and other toxic compounds harmful to the environment, plants, humans and animals. Too short a vegetation period of annual plants prevents the absorption of toxic substances, as it is in the case of long-growing trees. Ecological application of this ash in agriculture becomes more and more desired due to the increasing amount of biomass burned for energy purposes. This increase is in line with the global trend of reducing the use of fossil fuels and replacing them with renewable sources, including plant biomass fertilized with natural fertilizers^1–8^. The use of biomass in energy production is one of the global priorities in the field of pro-environmental activities. Burning biomass has a more favorable carbon dioxide balance than burning fossil fuels due to the absorption of CO_2_ by plants in photosynthesis. Both fly ash and biomass ash have similar chemical and mineral composition, exhibit a high pH and differ significantly from hard coal ash, which can contain composition of metal atoms^8–11^. The use of biomass for energy purposes and the volume of produced ash by its combustion is growing rapidly yearly^12, 13^. It is estimated that the part of biomass transferred to electricity, increased by 125% in 2020 as compared to 2010. With this increase the safe and sustainable use or disposal of the produced ash is a challenge. The development of technology of natural use of ash from plant biomass, for crop fertilizing purposes can be a promising solution, as it was demonstrated by Romanowska-Duda et al.^14^. An essential element in this fertilizing application is the fact that the content of biomass ash and its chemical composition can vary and may be related to vegetation type and soil conditions affecting growth and composition of plants, as well as their cultivation technology and storage. It can also change when the absorption of elements increases during long-term growth period of the same plant species, as the research of Vassiliev et al.^15^, Babayemi et al.^16^, Schiemenz et al.^17^, Michalik and Wilczyńska-Michalik^9^ and Lanzerstorfer^18^ show. Moreover, the amount of ash and its composition do not depend only on the plant species but also on its burned part. For example, Radacovska et al.^19^ showed that wood bark has more ash and lignin and less cellulose than wood. This necessitates the need to conduct comprehensive research on the stability of composition and fertilizing properties of ash from individual plant species, taking into account the environmental conditions and possibilities to replace or decrease artificial fertilization.

To date, most of the literature information relates to the use of burnt wood ash in the cultivation of forest plants^10, 20–23^. According to these researchers the application of wood ash can increase several nutrients quantity in forest soil, except nitrogen, change its pH, potentially stimulate microbial activity, improve tree growth and thus it can be used safely in forest land. However, especially in the case of ash obtained from long-growing woody plants, it may contain heavy metallic elements which may cause plant toxicity. Literature data on plant fertilization with ash from annual non-woody plants with a short vegetation period, including this from cereal straw, are much less numerous. According to Munawar et al.^13^ ash from annual plants does not contain heavy metals and can serve as a fertilizer causing a positive effect on soil fertility and plant growth. Higher soil fertility associated with a higher nutrient content due to the application of ash from burnt barley, wheat, rape seeds and some other species was shown by Piekarczyk et al.^24^. The addition of straw ash to soil significantly improved the dry weight of grains, shoots and roots of early rice^25^. Straw ash or straw biochar significantly increased also soil water retention, evapotranspiration and yield of spring barley and winter wheat in drought conditions, although it did not influence their grain yield^26^. It is also believed that ash from burned plants can be more useful as a fertilizer than sewage sludge due to its composition free of toxic compounds^27^. Data indicating the possibility to limit or eliminate artificial fertilization by ash from annual plant biomass are very scarce. The opportunity to reduce the artificial fertilization of energy plants by use of ash from Jerusalem artichoke combustion and biopreparations and possibility to enhance their growth and physiological activity was demonstrated by Romanowska-Duda et al.^14^. A similar effect was obtained after treating willow plants with algae^4^. According to our knowledge and available literature data, no research has yet been carried out on the use of natural ash from combusted sorghum in energy sorghum crops separately or together with digestate from biogas plants and bio-preparations, in order to show a possibility to reduce or eliminate artificial fertilization by use of this ash in closed production cycle of this crop. Moreover, majority of literature so far has focused on the use of single ash, mainly from particular wood species combustion, and no benefits have been demonstrated from combining this ash with digestates and biopreparations and the possibility of replacing chemical fertilization with them.

Our hypothesis is that use of sorghum ash as a fertilizer in subsequent energy sorghum crops may make closed and monocultural production of this species possible and thus circulation of nutrients, maintaining high soil fertility and limiting the use of harmful chemical fertilization and pesticides. To investigate this possibility, research was carried out on sorghum (*Sorghum bicolor* L.) ‘Rona 1’, a C4 crop, high yielding in Central Europe as compared to other varieties grown in subtropical areas^28^. Plants of this variety contain less indigestible fibre and can be used for food and energy purposes. Sorghum can be the leading energy-rich crop because due to the large biomass per ha (over 70 t ha^−1^), it can be a very attractive raw material for the production of large amounts of bioethanol (1000–4000 L of 95% ethanol and 15–30 tons of bagasse from 75–100 tons of biomass) and biogas (115 Nm^3^ from 1 ton of shoots) as well as briquettes, pellets, butanol and hydrogen which can be utilized for thermal, power or transport needs. Its biomass can be also torrefied to make biochar used as a valuable fuel for energy production and biofertilizer in crops, as our preliminary research showed. In addition, sorghum is a valuable raw material for the production of brooms, brushes, paper, wood-replacement boards and many other products, including preparations that regulate the growth of other organisms and are used to reduce weed infestation in crop^29^. In Polish climate, the green mass yield of this sorghum is 70–100 t ha^−1^. Because it grows well on poor, marginal and quarry soils (not intended for food production) and in drought conditions, needs of less fertilizers, grows rapidly, is easy for cultivation, lower cost of total fermentable sugars, and can be easily utilized for biofuel production, it can be an alternative as energy crop to maize, of which green mass yield is only 50 t ha^−1^ and it requires much more fertile and moist soil, that is difficult to provide in a changing climate^1, 5, 28, 30, 31^.

The object of the demonstrated studies was to evaluate the impact of natural sorghum ash, used separately or together with digestate from biogas plants, APOL-HUMUS and Stymjod, on development and physiological properties of sorghum plants under conditions of limited chemical fertilization with YaraMila Complex and to demonstrate the possibilities of reducing the recommended doses of synthetic fertilizers in different soil and climate conditions.

## Material and methods

### Plants, ash, waste, bio-preparations and soil

Sorghum (*Sorghum bicolor* L.) seeds of the Rona 1 variety were purchased in Kutno Sugar Beet Breeding Company (Poland). The multicomponent, chloride-free, synthetic fertilizer YaraMila Complex (Yara), containing macro- and microelements was purchased commercially. The ash was obtained by burning sorghum plants in own research. Before use it was sieved through 2 × 2 mm mesh. Digestate obtained from the digestion of corn grains to methane was produced in the Gamawind Sp. z o. o., Piaszczyna, Poland. APOL-HUMUS, a soil improver, was purchased from Poli-Farm Sp. z o.o., Poland. Stymjod, a mineral-nano-organic fertilizer, was provided by the manufacturer, PHU Jeznach Sp. J., Poland. The soil in field was assessed as sandy (Central Poland) and podzolic (Nord Poland) and of little use for food production. The parameters of these materials are shown in Table 1.

**Table 1:** Quantity of macro and microelements in ash from sorghum biomass, soil before and after fertilization with it (4 t ha^−1^), digestate from biogas plant, and biopreparations.

Assessed material	N	P	K	Ca	Mg	Fe	Mn	Cu	Zn	B	Dry mass	pH
	[mg kg^−1^ dry weight]										[%]	
Ash from sorghum	0.48 a ± 0.15	14,220 d ± 8.3	163,000 d ± 8,2	38,430 d ± 8.9	9961 d ± 8.0	2330 d ± 8.0	148 d ± 4.0	29.7 e ± 1.0	1148 d ± 9.0	61.9 d ± 1.1	89.6 b ± 1.3	12.0 b ± 0
Podzolic soil not fertilized	1.03 c ± 0.15	945 b ± 10.0	3829 b ± 11.1	25,945 b ± 11.3	1582 b ± 10.3	661 b ± 9.9	45.6 b ± 3.1	24.3 c ± 1.1	30.0 b ± 1.0	63.3 b ± 1.2	25.6 a ± 1.3	5.6 a ± 0
Podzolic soil + sorghum ash	1.10 d ± 0.16	1225 c ± 11.1	5139 c ± 11.4	30,566 c ± 11.3	2279 c ± 10.2	987 c ± 10	57.9 c ± 3.9	26.2 d ± 1.0	36.5 c ± 1.1	64.2 c ± 1.0	25.9 a ± 1.0	5.8 a ± 0
Sandy soil not fertilized	0.81 b ± 0.16	401 a ± 12.2	3123 a ± 10.3	22,456 a ± 10.2	999 a ± 9.5	600 a ± 9.2	39.5 a ± 4.0	13.5 a ± 1.0	14.5 a ± 0.8	30.3 a ± 0.9	26.1 a ± 1.0	6.2 b ± 0
Sandy soil + sorghum ash	1.01 c ± 0.16	963 b ± 12.5	3858 b ± 12.9	26,014 b ± 11.6	1606 b ± 10.1	660 b ± 9.9	44.2 b ± 4.0	19.1 b ± 0.8	29.5 b ± 0.9	59.9 b ± 0.9	26.5 a ± 1.0	6.3 b ± 0
	[m L^−1^]											
Digestate	2459 c ± 0.8	274 b ± 0.1	998 b ± 0.3	302 a ± 0.0	119 b ± 0.2	9.1 a ± 0.1	0.326 a ± 0.0	0.177 a ± 0.0	0.979 a ± 0.0	3.367 b ± 0.4	1.5 a ± 0.1	7.6 a ± 0
Stymjod^a^	1231 b ± 0.0	6652 c ± 0.1	62,720 c ± 0.1	943 c ± 0.0	11,570 c ± 0.1	18.9 b ± 0,2	886 c ± 0.1	682 c ± 0.0	1476 c ± 0.2	576 c ± 0.0	–	–
Apol-humus^b^	15.20 a ± 0.1	15.7 a ± 0.1	20.0 a ± 0.1	466 b ± 0.0	71 a ± 0.0	142 c ± 0.3	5.98 b ± 0.0	0.89 b ± 0.0	2.42 b ± 0.0	0.94 a ± 0.0	–	12.0 b ± 0

^a,b^Manufacturer's data; pH: ash 12.0, soil 5.1, non-centrifuged waste 7.6, Apol-humus 12.0. The data with the same letters within columns and separately for [mg kg^−1^ dry weight] and [mg L^−1^] parameters are not significantly different, according to Newman–Keuls multiple range test at an alpha level of 0.05. The values are the mean ± SD of three replicates*.*

### Treatments and experimental design

Research was performed in diverse weather and climate conditions of Central (Skierniewice, 51°57′010″N, 20°08′030″E) and Northern Poland (Piaszczyna, 54°01′21″N, 17°10′19″E), where plants were cultivated in the field, in the sandy and podzolic soils, respectively, in order to show the effects of the used biofertilizers in different climate and soil conditions and water availability. In Skierniewice (Central Poland), the summer temperature ranges from 20 to 32 °C, yearly precipitation is about 528 mm and more sunny days and dry air occurs. In Piaszczyna area (Northern Poland) temperature ranges usually from 11 to 21 °C, with an average annual precipitation of about 655 mm and humid air from the Baltic Sea is often observed. All treatments and plant assessments in both localizations were made at the same dates.

**Figure 1:**
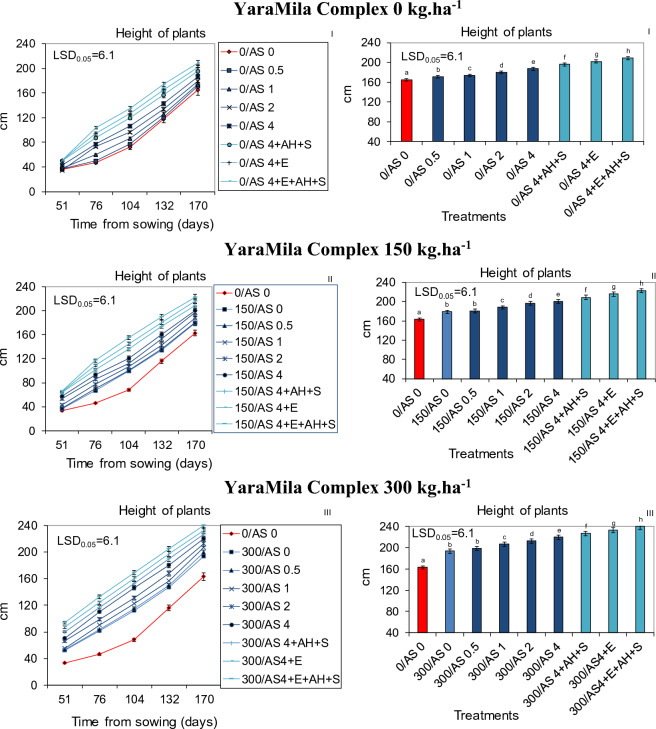
Dynamics of growth and the finishing height of sorghum plants cultured in podzolic soil in North Poland and fertilized with YaraMila Complex amounts of: 0 (I), 150 (II) and 300 kg ha^−1^ (III) and within each amount, with the sorghum ash (AS 0–4.0 t ha^−1^) applied separately or together with APOL-HUMUS (AH; 10 L ha^−1^), Stymjod 1.5% (S; 5 L ha^−1^) or biogas plant digestate (E; 30 m^3^ ha^−1^), The LSD was calculated at the significance level of p = 0.05. The means with the same letters are not significantly different, according to Newman–Keuls multiple range test at an alpha level of 0.05. Error bars show mean ± SD of three independent replicates.

**Figure 2:**
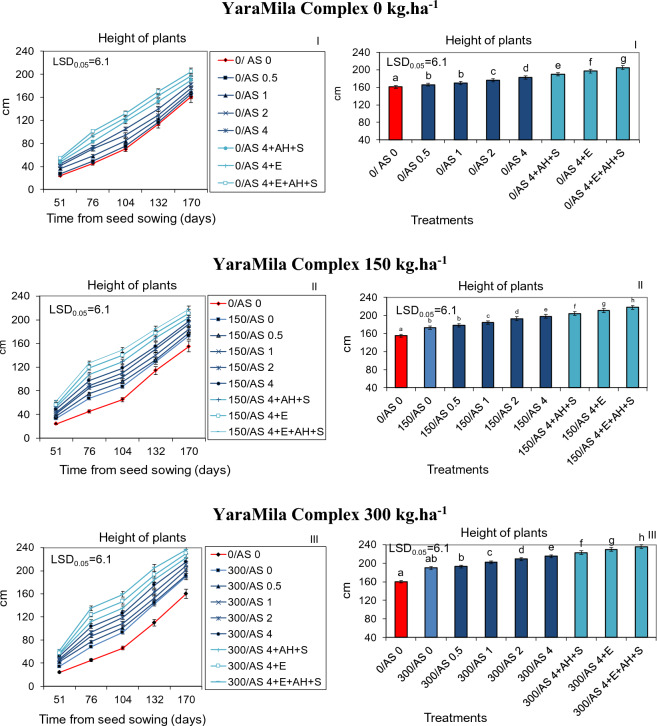
Dynamics of growth and height at harvest time of sorghum plants cultured in poor sandy soil in central Poland and fertilized with YaraMila Complex amounts of: 0 (I), 150 (II) and 300 kg ha^−1^ (III) and within each amount, with the sorghum ash (AS 0–4.0 t ha^−1^) applied separately or together with APOL-HUMUS (AH; 10 L ha^−1^), Stymjod 1.5% (S; 5 L ha^−1^) or biogas plant digestate (E; 30 m^3^ ha^−1^). The LSD was calculated at the significance level of p = 0.05. Means with the same letters are not significantly different, according to Newman–Keuls multiple range test at an alpha level of 0.05. Error bars show mean ± SD of three independent replicates.

**Figure 3:**
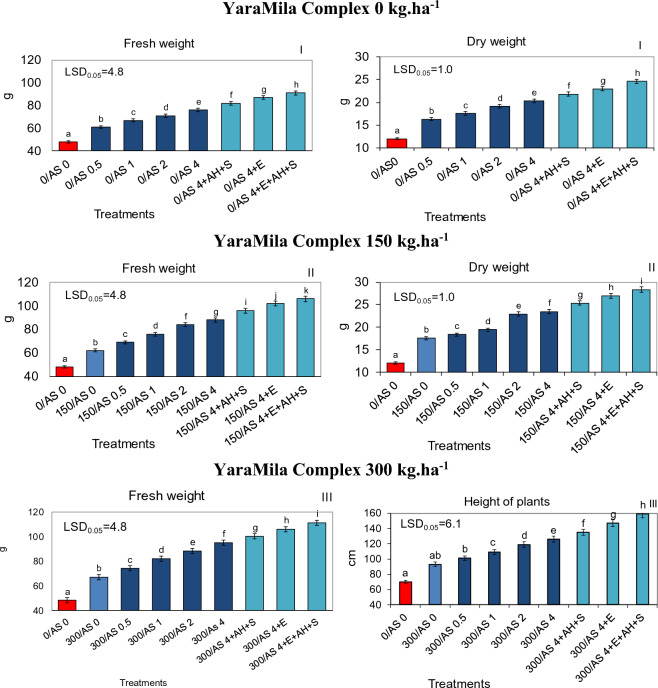
Fresh and dry weight, calculated per one sorghum plant, cultured in poor sandy soil in central Poland and fertilized with YaraMila Complex amounts of 0 (I), 150 (II) and 300 kg ha^−1^ (III) and within each amount, with the sorghum ash (AS 0–4.0 t ha^−1^) applied alone or together with APOL-HUMUS (AH; 10 L ha^−1^), Stymjod 1.5% (S; 5 L ha^−1^) or biogas plant digestate (E; 30 m^3^ ha^−1^). The LSD was calculated at the significance level of p = 0.05. The means marked with the same letters are not significantly different, according to Newman–Keuls multiple range test at an alpha level of 0.05. Error bars show mean ± SD of three independent replicates.

The study was conducted in three separate blocks performed simultaneously in both localizations, in which the soil was enriched in April with the artificial fertilizer YaraMila Complex at doses of 0, 150 and 300 kg ha^−1^. The dose of 300 kg ha^−1^ was selected on the basis of previous own research as beneficial for sorghum, Jerusalem artichoke and corn as well as apple trees and field vegetables and in accordance with the manufacturer's recommendation for horticultural crops, although there is no other literature data on the use of this fertilizer in sorghum. Reducing this dose to 150 and 0 kg ha^−1^ made it possible to check the possibility of replacing YaraMila Complex with ash, partially or completely. Then, within each of this artificial fertilizer doses (blocks), the soil in each separate field plot (in Central and Northern Poland) was additionally enriched with ash from burnt sorghum in amounts of 0.5, 1, 2 and 4 t ha^−1^, to check their effectiveness in replacing synthetic fertilizer, taking into account the adverse effect of higher doses in previous author's studies. In the subsequent experimental variants, the plots treated with YaraMila Complex (0–300 kg ha^−1^) and ash at the dose of 4 t ha^−1^ were also supplementary fertilized with digestate from corn grain digestion to methane (30 m^3^ ha^−1^) and APOL-HUMUS (10 L ha^−1^)^3^. In addition, double foliar application of Stymjod (5 L concentrate ha^−1^), at a 2-week interval in July, was used. YaraMila Complex, ash, digestate and APOL-HUMUS were mixed with the soil after their application. The experimental variants are presented in Figs. 1, 2, 3 and Tables 2, 3, 4.

**Table 2:** Gas exchange and index of chlorophyll content in sorghum plants, fertilized with YaraMila Complex amounts of 0, 150 and 300 kg ha^−1^ and within each amount, with the sorghum ash (AS 0–4.0 t ha^−1^) used separately or together with APOL-HUMUS (AH; 10 L ha^−1^), Stymjod (S; 5 L ha^−1^) or digestate from biogas plant (E; 30 m^3^ ha^−1^).

Applied waste	Net photosynthesis [µm CO_2_ m^−2^ s^−1^]	Transpiration [mmol H_2_O m^−2^ s^−1^]	Stomatal conductance [mmol m^−2^ s^−1^]	Intercellular concentration of CO_2_ [µmol CO_2_ air mol^−1^]	Index chlorophyll content [SPAD]
YaraMila Complex 0 kg ha^−1^					
AS 0	4.0 a ± 0.4	0.61 a ± 0.2	164 a ± 7.7	345 m ± 11.2	20.1 a ± 0.3
AS 0.5	5.5 bc ± 0.5	0.66 b ± 0.3	274 d ± 7.9	327 kl ± 11.2	20.6 b ± 0.3
AS 1	5.7 bcd ± 0.5	0.78 c ± 0.3	278 d ± 7.8	316 jk ± 11.0	21.5 c ± 0.3
AS 2	6.7 def ± 0.5	0.90 e ± 0.2	296 e ± 8.0	305 i ± 11.1	22.4 ef ± 0.3
AS 4	7.0 f ± 0.6	0.96 f ± 0.3	311 f ± 8.4	294 hi ± 9.2	22.9 gh ± 0.4
AS4 + AH10 + S	7.9 ghl ± 0.5	1.02 g ± 0.4	321 g ± 8.3	263 fg ± 9.4	23.4 ij ± 0.4
AS 4 + E 30	8.5 ig ± 0.5	1.12 h ± 0.4	330 gh ± 8.7	269 g ± 9.7	23.7 ijk ± 0.4
AS4 + E30 + AH10 + S	9.3 k ± 0.6	1.20 i ± 0.4	336 h ± 8.9	256 f ± 9.6	24.0 kl ± 0.3
YaraMila Complex 150 kg ha^−1^					
AS 0	5.2 b ± 0.4	0.73 c ± 0.2	187 b ± 8.0	314 j ± 9.9	20.6 b ± 0.2
AS 0.5	5.9 cd ± 0.4	1.20 i ± 0.3	296 e ± 8.4	300 i ± 9.9	21.5 c ± 0.2
AS 1	6.3 de ± 0.5	1.32 j ± 0.3	362 i ± 8.7	283 h ± 10.1	22.0 de ± 0.4
AS 2	7.7 gh ± 0.5	1.46 k ± 0.4	389 k ± 8.9	256 f ± 10.3	22.5 fg ± 0.4
AS 4	8.3 hij ± 0.6	1.81 m ± 0.5	400 l ± 8.9	234 e ± 9.9	23.3 hi ± 0.4
AS4 + AH10 + S	9.4 k ± 0.6	1.94 n ± 0.4	415 m ± 8.8	204 d ± 10.3	23.8 jk ± 0.3
AS 4 + E 30	9.5 k ± 0.5	2.11 o ± 0.4	426 n ± 9.0	182 c ± 9.4	24.4 l ± 0.3
AS4 + E30 + AH10 + S	11.2 m ± 0.7	2.20 p ± 0.4	438 o ± 9.1	160 b ± 9.4	25.0 mn ± 0.4
YaraMila Complex 300 kg ha^−1^					
AS 0	6.3 de ± 0.4	0.84 d ± 0.2	236 c ± 8.2	285 h ± 10.5	21.7 cd ± 0.2
AS 0.5	7.3 fg ± 0.5	0.95 f ± 0.2	302 ef ± 8.5	263 fg ± 10.4	22.2 ef ± 0.3
AS 1	7.8 gh ± 0.5	1.49 k ± 0.2	374 j ± 8.7	256 f ± 10.1	22.6 fg ± 0.3
AS 2	8.2 hi ± 0.6	1.63 l ± 0.4	405 l ± 8.9	223 e ± 9.3	23.3 hi ± 0.4
AS 4	8.9 jk ± 0.6	1.95 n ± 0.3	416 m ± 8.9	192 cd ± 9.3	24.6 m ± 0.4
AS4 + AH10 + S	10.2 l ± 0.7	2.10 o ± 0.4	436 o ± 9.1	181 c ± 9.3	25.2 n ± 0.4
AS 4 + E 30	10.7 lm ± 0.6	2.21 p ± 0.4	447 p ± 9.2	161 b ± 9.2	25.7 o ± 0.4
AS4 + E30 + AH10 + S	11.9 n ± 0.7	2.33 r ± 0.4	458 r ± 9.1	137 a ± 9.2	26.6 p ± 0.4

The means marked with the same letters within a column are not significantly different, according to Newman–Keuls multiple range test at an alpha level of 0.05. The values are the mean ± SD of three replicates.

**Table 3:** Activity of the selected enzymes in sorghum plants fertilized with YaraMila Complex amounts of 0, 150 and 300 kg ha^−1^ and within each amount, with the sorghum ash (AS 0–4.0 t ha^−1^) used separately or together with APOL-HUMUS (AH; 10 L ha^−1^), Stymjod (S; 5 L ha^−1^) or digestate from biogas plant (E; 30 m^3^ ha^−1^).

Treatment	Phosphatase: (pH = 6.0) [U g^−1^ f.w.]	Phosphatase (pH = 7.5) [U g^−1^ f.w.]	RNAse (U g^−1^ f.w.)	Total dehydrogenases [mg formazan × g leaf^−1^]
YaraMila Complex 0 kg ha^−1^				
AS 0	0.50 a ± 0.16	0.15 a ± 0.01	2.35 a ± 0.09	0.42 a ± 0.02
AS 0.5	0.61 bc ± 0.17	0.24 bc ± 0.02	3.17 c ± 0.09	0.60 c ± 0.02
AS 1	0.64 de ± 0.17	0.26 cd ± 0.02	3.26 cd ± 0.09	0.64 d ± 0.02
AS 2	0.68 fg ± 0.18	0.28 d ± 0.02	3.42 ef ± 0.10	0.69 e ± 0.02
AS 4	0.73 h ± 0.17	0.31 e ± 0.02	3.62 gh ± 0.10	0.75 f ± 0.03
AS4 + AH10 + S	0.78 ij ± 0.19	0.34 fg ± 0.02	3.73 ijk ± 0.09	0.79 gh ± 0.03
AS 4 + E 30	0.80 jkl ± 0.19	0.36 gh ± 0.02	3.83 kl ± 0.10	0.81 hi ± 0.03
AS4 + E30 + AH10 + S	0.83 m ± 0.18	0.39 ij ± 0.02	3.95 mn ± 0.09	0.85 jk ± 0.02
YaraMila Complex 150 kg ha^−1^				
AS 0	0.59 b ± 0.17	0.23 b ± 0.1	2.49 b ± 0.09	0.56 b ± 0.02
AS 0.5	0.66 ef ± 0.18	0.27 d ± 0.01	3.31 d ± 0.09	0.69 e ± 0.02
AS 1	0.73 h ± 0.16	0.31 e ± 0.1	3.52 fg ± 0.10	0.76 fg ± 0.02
AS 2	0.76 i ± 0.17	0.33 ef ± 0.02	3.69 ij ± 0.10	0.81 hi ± 0.03
AS 4	0.78 ij ± 0.18	0.34 fg ± 0.02	3.83 kl ± 0.10	0.83 ij ± 0.03
AS4 + AH10 + S	0.81 kl ± 0.18	0.37 hij ± 0.03	3.96 mn ± 0.10	0.87 k ± 0.03 l
AS 4 + E 30	0.83 m ± 0.19	0.39 ij ± 0.02	3.98 no ± 0.10	0.89 l ± 0.03
AS4 + E30 + AH10 + S	0.87 o ± 0.20	0.42 k ± 0.03	4.16 pr ± 0.11	0.93 m ± 0.04
YaraMila Complex 300 kg ha^−1^				
AS 0	0.63 cd ± 0.18	0.27 d ± 0.01	3.17 c ± 0.09	0.63 cd ± 0.03
AS 0.5	0.69 g ± 0.19	0.32 ef ± 0.01	3.36 de ± 0.10	0.71 e ± 0.04
AS 1	0.79 jk ± 0.19	0.34 fg ± 0.02	3.67 hi ± 0.10	0.81 hi ± 0.03
AS 2	0.82 lm ± 0.21	0.36 gh ± 0.03	3.79 jkl ± 0.11	0.85 jk ± 0.03
AS 4	0.84 mn ± 0.19	0.38 hij ± 0.02	3.98 no ± 0.11	0.89 l ± 0.03
AS4 + AH10 + S	0.86 no ± 0.21	0.41 k ± 0.02	4.12 pr ± 0.10	0.93 m ± 0.2
AS 4 + E 30	0.88 o ± 0.21	0.42 k ± 0.03	4.19 r ± 0.10	0.95 m ± 0.03
AS4 + E30 + AH10 + S	0.91 p ± 0.20	0.46 l ± 0.03	4.30 s ± 0.11	0.99 n ± 0.04

The data marked with the same letters within a column are not significantly different. according to Newman–Keuls multiple range test at an alpha level of 0.05. The values are the means ± SD of three replicates.

**Table 4:** Percentage of pathogenic fungi on the infested leaves in relation to total isolates and the number of infected plants. as effected by sorghum plant fertilization with YaraMila Complex amounts of 0, 150 and 300 kg ha^−1^ and within each amount, with the sorghum ash (AS 0–4.0 t ha^−1^) used separately or together with APOL-HUMUS (AH; 10 L ha^−1^), Stymjod (S; 5 L ha^−1^) or digestate from biogas plant (E; 30 m^3^ ha^−1^).

Pathogenic fungi	Dose of YaraMila complex [kg ha^−1^]	Fertilization with ash (AS 0–4 t ha^−1^) or ash (4 t ha^−1^) digestate (E), APOL-HUMUS (AH) and Stymjod (S)				
		AS 0	AS 1	AS 2T 2	AS 4	AS4 + E + AH + S
Percentage of pathogenic fungi on the infested leaves in relation to total isolates						
*Colletotrichum* spp.	0	6.1 k ± 0.3	6.0 jk ± 0.3	5.5 gh ± 0.3	5.0 e ± 0.2	4.3 d ± 0.2
	150	5.8 ij ± 0.3	5.6 hi ± 0.3	5.2 ef ± 0.3	4.5 d ± 0.2	3.5 b ± 0.2
	300	5.5 gh ± 0.3	5.3 fg ± 0.3	5.1 ef ± 0.3	4.0 c ± 0.2	2.9 a ± 0.2
*Exserohilum turcicum*	0	4.3 j ± 0.3	4.0 i ± 0.3	3.7 h ± 0.3	3.5 gh ± 0.2	3.0 de ± 0.2
	150	4.0 i ± 0.3	3.7 h ± 0.3	3.2 ef ± 0.2	2.8 d ± 0.2	2.3 c ± 0.2
	300	3.3 fg ± 0.3	3.0 de ± 0.3	2.4 c ± 0.2	1.5 b ± 0.3	0.0 a ± 0.0
*Fusarium* spp*.*	0	2.2 i ± 0.1	2.0 h ± 0.1	1.6 fg ± 0.1	1.4 e ± 0.1	1.2 d ± 0.1
	150	2.0 h ± 0.1	1.7 g ± 0.1	1.4 e ± 0.1	1.2 d ± 0.1	1.0 c ± 0.1
	300	1.7 g ± 0.1	1.5 ef ± 0.1	1.2 d ± 0.1	0.8 b ± 0.1	0.5 a ± 0.1
*Alternaria* sp*.*	0	5.3 i ± 0.2	5.0 k ± 0.1	4.4 hi ± 0.1	3.8 f ± 0.1	2.5 c ± 0.1
	150	5.0 k ± 0.2	4.8 jk ± 0.1	4.0 fg ± 0.1	3.1 d ± 0.1	2.3 b ± 0.1
	300	4.6 ij ± 0.1	4.2 h ± 0.2	3.4 e ± 0.1	2.5 c ± 0.1	1.8 a ± 0.1
*Rhizoctonia* spp.	0	1.3 g ± 0.1	1.1 f ± 0.1	0.6 d ± 0.1	0.5 cd ± 0.1	0.4 c ± 0.1
	150	1.1 f ± 0.1	0.8 e ± 0.1	0.5 cd ± 0.1	0.4 c ± 0.1	0.2 b ± 0.1
	300	0.6 d ± 0.1	0.4 c ± 0.1	0.2 b ± 0.1	0.2 b ± 0.1	0.0 a ± 0.0
*Pythium* sp.	0	1.5 h ± 0.1	1.5 cd ± 0.1	1.1 fg ± 0.1	0.8 e ± 0.1	0.0 a ± 0.0
	150	1.2 g ± 0.1	1.0 f ± 0.1	0.5 cd ± 0.1	0.4 c ± 0.1	0.0 a ± 0.0
	300	0.5 cd ± 0.1	0.4 c ± 0.1	0.2 b ± 0.1	0.0 a ± 0.0	0.0 a ± 0.0
*Peronosclerospora sorghi*	0	2.9 i ± 0.2	2.6 h ± 0.1	2.0 f ± 0.1	1.5 e ± 0.1	0.7 c ± 0.1
	150	2.6 h ± 0.2	2.4 g ± 0.1	1.6 e ± 0.1	0.8 c ± 0.1	0.5 b ± 0.1
	300	2.4 g ± 0.2	1.5 e ± 0.1	1.0 d ± 0.1	0.5 b ± 0.1	0.0 a ± 0.0
Percent of infected plants						
% of infested plants	0	12.5 l ± 0.7	11.6 k ± 0.6	10.4 i ± 0.6	9.0 g ± 0.6	7.5 e ± 0.5
	150	11.0 j ± 0.7	10.3 hi ± 0.6	8.2 f ± 0.6	7.5 e ± 0.6	5.0 c ± 0.5
	300	9.8 h ± 0.6	8.5 fg ± 0.6	6.0 d ± 0.6	4.0 b ± 0.6	3.1 a ± 0.5

The data marked with the same letters within particular pathogen species or within % of infected plants are not significantly different according to Newman–Keuls multiple range test at an alpha level of 0.05. The values are the means ± SD of three replicates.

In May, sorghum seeds were sown into the soil (enriched with the aforementioned fertilizers). The 175 seeds per plot (3 × 3 m) at 35 × 12 cm distance were used according to practical recommendations. Non- fertilized plants were used as control. Dosages of used YaraMila Complex, digestate from biogas plant, APOL-HUMUS and Stymjod were chosen on the basis of previous studies^14^.

### Assessments of plant physiological activity, growth and chemical properties

Soil and plant samples for testing were collected and evaluated according to methodologies developed on the basis of national standards^32^ and previous research, which are in force at The National Institute of Horticultural Research in Poland and have been used in previous studies and international publications^3, 4, 14^. The effects of treatments in field trials were evaluated by monthly height measurements of plants throughout the vegetative period while physiological activity in July and yield and energy properties of biomass in autumn^4^. Evaluations of gas exchange, index of chlorophyll content, enzyme activity, infestation by pathogenic fungi and nutrient content were made on fully grown leaves located in the upper part of the plant. From each repetition, 10 plants of a given variant were randomly selected and one leaf was taken from each of them, intended for mycological and physiological activity assessment. All these leaves, together with those for nutrient estimation (100 g), were taken at the end of July at temperature of 25–30 °C, in sunshine and air humidity of 50–60%.

The fresh (directly after harvest) and dry (dried at 130 °C for 3 days) weights of plants were evaluated at the end of November, taking into account 5 plants and calculated for one plant as average for each investigational variant^33^.

Gas exchange in leaves (net photosynthesis, transpiration, stomatal conductance and concentration of intercellular CO_2_), was measured using the apparatus TPS-2 Portable Photosynthesis System (Amesbury, USA)^14^.

Index of chlorophyll content in leaves was measured with the apparatus SPAD-502 (Japan)^34^.

Activities of both phosphatase: pH 6; EC 3.1.3.2 and pH 7.5; EC 3.1.3.1 (U g^−1^ (FM) min^−1^), and RNase (EC 3.1.27.5) (U g^−1^ (FM) min^−1^) were assessed according to the methods elaborated by Knypl and Kabzinska^35^.

Activity of dehydrogenases (EC 1.1.1.-) was evaluated using the method elaborated by Górnik and Grzesik^36^. Shimadzu™ UVmini-1240 Model Spectrophotometer and a wavelength of 480 nm was used for measurement of formazan amount.

The pathogenic fungi species and their quantity on plants were estimated after harvest of infected leaves (one from each of 10 plants) and their incubation in humid cameras for 7 days. After that they were moved from the leaves into selective media. The identification of particular fungi species and their amount on media were documented using identification keys and light microscopy (Leica). The percentage of plants infested with pathogenic fungi was assessed considering 175 plants in a field plot^37–39^.

Qualitative and quantitative composition of elements in sorghum ash, APOL-HUMUS Stymjod, leaves and in soil, before and after ash application, was evaluated in the licensed laboratory at National Institute of Horticultural Research (Skierniewice, Poland). The content of selected macro- and micronutrients in the leaves was assessed using a PerkinElmer OPTIMA 2000™ ICP optical emission spectrometer (PerkinElmer Inc., USA). The content of total nitrogen in leaves was studied by the Kjeldahl method after mineralization in concentrated sulphuric acid with the addition of a catalyst^40^. Energy values of plants were evaluated by licensed laboratory (Carbochem, Poland)^14^.

### Statistical analysis

The research was performed in three following years (series) in central and north Poland and in three replicates for each investigational variant. All replicates and examined variants were set up in randomized block design. The obtained results, given as means from replicates and series, were processed using analysis of variance (ANOVA I), by Statistica 12. The data presented in tables and figures were grouped employing the Newman–Keuls multiple range test at the α = 0.05 significance level.

## Results

### Determination of the effect of natural ash and limited artificial fertilization on the growth of sorghum

The dynamics of growth, biomass yield and physiological activity of plants increased in proportion to the increased doses of both fertilizers applied simultaneously, YaraMila Complex from 0 to 300 kg ha^−1^ and ash from 0 to 4 t ha^−1^. The ash at the dose of 4 t ha^−1^ was most beneficial for sorghum development regardless of artificial fertilization level. The positive influence of both fertilizers, YaraMila Complex and natural ash, on sorghum growth and physiological properties was intensified by additional supplementary soil treatment with APOL-HUMUS (10 L ha^−1^) or digestate from the biogas plant (30 m^3^ ha^−1^) and also by leaf double spraying with Stymjod (5 L ha^−1^). Therefore, the most beneficial effect on the growth and yield of biomass was caused by the fertilization with Yara Mila Complex (300 kg ha^−1^), ash from sorghum (4 t ha^−1^), digestate from biogas plant (30 m^3^ ha^−1^), APOL-HUMUS (10 L ha^−1^) and Stymjod (5 L ha^−1^). Moreover, the combined use of the favorable ash dose of 4 t ha^−1^ and smaller quantities of YaraMila Complex (0 or 150 kg ha^−1^) increased the dynamics of plant growth and yield of biomass to the same extent as the twice higher doses of this artificial fertilizer applied alone (respectively 150 or 300 kg ha^−1^) (Figs. 1, 2, 3).

The relationships between the discussed plant treatments and sorghum development were similar in sandy and podzolic soil carried out, respectively, in central Poland, with less favorable climate and soil conditions, and in the north near the Baltic Sea. In all experimental variants due to the more favorable soil and climate conditions, plants grown in the northern part of the country grew more intensively than in the lower quality soil in the central Poland, however the relationships indicated above between the doses of fertilizers used and development were maintained (Figs. 1, 2).

### Effect of natural ash and limited artificial fertilization on physiological activity and infestation by pathogenic fungi

The above described relationships between plant growth or biomass yield and YaraMila Complex and ash doses were also demonstrated by the studies of gas exchange and chlorophyll content index. Higher dosages of this artificial fertilizer and ash, together with digestate and biopreparations, in the tested configurations, caused a proportional increase in index of chlorophyll content, net photosynthesis, transpiration, stomata conductance and decrease in intercellular CO_2_ concentration. Similarly, as in the case of plant growth assessment, the sorghum ash used in optimal amounts together with smaller amounts of YaraMila Complex (0 or 150 kg ha^−1^) had a more favorable impact on the tested physiological activities than this fertilizer applied alone but in two times larger dosages (Figs. 1, 2, 3, Table 2).

The plant fertilization with YaraMila Complex and sorghum ash resulted also in increased activity of phosphatases (pH 6; EC 3.1.3.2 and pH 7.5), RNase and total dehydrogenases. Their activities enhanced as the doses of YaraMila Complex and ash increased in all experimental variants, with 4 t of ash per ha being the most effective, regardless of the amount of the synthetic fertilizer used. Changes in the activity of these enzymes indicated also that the applied optimal doses of sorghum ash make it possible to reduce the recommended doses of YaraMila Complex from 150 to 0 and from 300 to 150 kg ha^−1^ (Table 3).

Sorghum plants grown in all conditions was contaminated with pathogenic fungi belonging to seven species. The application of YaraMila Complex and sorghum ash caused a reduction in plant infection by pathogenic microflora, in proportion to the increasing doses of both fertilizers. Their supplementation with digestate from biogas plant, APOL-HUMUS and Stymjod additionally positively influenced the health of plants. The application of ash at the mentioned above optimal amount and lesser quantity of YaraMila Complex (0 or 150 kg ha^−1^) caused a reduction of infection with pathogenic fungi to a similar extent as the twice higher dose of synthetic fertilizer applied separately, 150 or 300 kg ha^−1^, respectively (Figs. 1, 2, 3, Tables 2, 3, 4).

### Effect of ash and limited artificial fertilization on element content and energy properties

The application of YaraMila Complex and sorghum ash caused a slight increase in the content of elements in leaves in proportion to the used doses of fertilizers (Table 5). Fertilizing the plants with the increased dosages of the tested artificial fertilizer and ash also improved a little biomass energy properties, and caused a reduction of the ash content in the burnt plant mass, as it is demonstrate in Table 6.

**Table 5:** Quantity of macro and microelements in leaves of sorghum cultured in soil, non-fertilized (0) or enriched with YaraMila Complex (YMC) amounts of 0–300 kg ha^−1^, together with sorghum ash (AS 4 t ha^−1^).

YMC dose	Ash [t ha^−1^]	N	P	K	Ca	Mg	Na	S.SO_4_	Fe	Mn	Cu	Zn	B
		[%]	[mg kg^−1^ d.w.]										
0	0	2.02 a ± 0.09	4789 a ± 88	20,490 a ± 540	7727 a ± 140	2590 a ± 43	127 a ± 6	1423 a ± 19	299 a ± 14	20.5 a ± 1	13.4 a ± 1	32.3 a ± 1.2	18.9 a ± 1.2
0	AS 4	2.04 a ± 0.09	4849 a ± 99	21,559 b ± 525	8159a ± 123	2630 ab ± 43	130 a ± 6	1433 a ± 18	300 a ± 14	20.9 a ± 1	13.9 a ± 1	32.8 a ± 1.2	19.2 a ± 1.1
150	AS 4	2.07 ab ± 0.09	4989 b ± 99	21,589 b ± 510	8169 a ± 150	2643 b ± 40	132 a ± 6	1434 a ± 20	308 a ± 14	21.0 a ± 1	14.0 a ± 1	32.9 a ± 1.1	19.3 a ± 1.1
300	AS 4	2.12 b ± 0.09	5020 b ± 99	21,621 b ± 490	8184 a ± 150	2655 b ± 40	133 a ± 6	1439 a ± 20	311 a ± 15	21.1 a ± 1	14.2 a ± 1	33.0 a ± 1.1	19.3 a ± 1.1

The data marked with the same letters within particular evaluated element are not significantly different. according to Newman–Keuls multiple range test at an alpha level of 0.05. The values are the mean ± SD of three replicates.

**Table 6:** Energy properties of sorghum cultured in soil non-fertilized (Control) and fertilized with YaraMila Complex (0, 150, 300 kg ha^−1^) together with sorghum ash (4.0 t ha^−1^), APOL-HUMUS (10 L ha^−1^), Stymjod 1.5% (5 L ha^−1^) and digestate from biogas plant (30 m^3^ ha^−1^).

Evaluated properties	Research method	Unit of measure	Control	YaraMila dose (kg ha^−1^)		
				0	150	300
Analytical state						
Analytical humidity	PN-G-04511;1980	%	5.21 a ± 0.16	5.42 b ± 0.16	5.72 c ± 0.16	6.37 d ± 0.16
Ash	PN-ISO 1171:2002	%	10.1 d ± 0.23	9.24 c ± 0.21	8.01 b ± 0.21	7.44 a ± 0.21
Heat of combustion	PN ISO 1928:2002	kJ kg^−1^	16,629 a ± 25.8	16,659 b ± 25.9	16,916 c ± 26.3	17,180 d ± 26.2
Working state						
Transient humidity	PN-G-04511;1980	%	50.92 a ± 2.2	50.82 a ± 2.1	50.12 a ± 2.1	49.43 a ± 2.1
Total humidity	PN-G-04511;1980	%	53.51 d ± 0.10	53.40 c ± 0.10	53.05 b ± 0.10	52.90 a ± 0.10
Ash	PN-ISO 1171:2002	%	4.99 d ± 0.17	4.65 c ± 0.17	4.10 b ± 0.17	3.87 a ± 0.16
Calorific value	PN ISO 1928:2002	kJ kg^−1^	6369 a ± 20.1	6419 b ± 20.2	6568 c ± 20.2	6591 d ± 20.2

The means with the same letters within particular evaluated parameter are not significantly different. according to Newman–Keuls multiple range test at an alpha level of 0.05. The values are the mean ± SD of three replicates.

## Discussion

Increasing production of plant biomass for energy purposes requires new technologies of its sustainable use making reduction of synthetic fertilization and environmental pollution by agricultural chemicals possible. The presented research demonstrates a new technology showing that natural sorghum ash, used alone or together with digestate and bio-preparations, can serve as ecological fertilizer enhancing plant development and their yielding regardless of environments and its use can replace or decrease the presently recommended dosages of chemical fertilizers. The elaborated technology also enables the closed production of particular plant species and closed circulation of nutrients between biomass and soil possible, maintaining its high fertility. This ash, used alone or enriched with digestate and biopreparations, can be used for fertilization of sorghum ‘Rona 1’ which can be a forward-looking and high-yielding energy plant in Central Europe with multidirectional use in energy industry^28, 41–44^. This plant develops well in poor, dry and saline soil and high temperature conditions, which occur in the changing climate^28^. The presented results extend the existing knowledge on the possibility of using ash in plant fertilization, which so far has focused on the use of these wastes, mainly from wood combustion, individually and no benefits have been demonstrated from combining this ash with digestates and bio preparations and the possibility of replacing chemical fertilization with them. The presented studies can also be the basis for further research on ensuring the balance of nutrients between plants and soils with fertilizers.

The results show a stimulating impact of natural sorghum ash on the tested plant development and biomass yield. This was due to the high content of macro- and microelements necessary for plant development in this ash, as stated also by Wierzbowska et al.^45^ who assessed the impact of ash from energy willow and Pennsylvania fanpetals on soil properties and yield of willow grown as an energy crop. Phosphorus, potassium and magnesium taken up by plants from the tested ash were similar as from mineral salts, as it was mentioned also by Schiemenz et al.^17^ who used the waste from combusted biomass of different crops. This fertilization with ash increased also the amount of micronutrients and phytoavailable forms of phosphorus, potassium and magnesium in soil. The presented results show that the addition of sorghum ash (preferably 4 t ha^−1^) to each tested dose of YaraMila Complex (0, 150, 300 kg ha^−1^) increases the fertilizing potential of this synthetic fertilizer. This treatment enhanced the content of macro- and microelements in soil, which resulted in higher dynamics of plant growth, their yielding, and increased physiological activity and energy value of biomass, which strictly depended on the applied amounts of the tested fertilizers. This indicates that natural sorghum ash can be used as an ecological fertilizer as it has a positive effect on soil fertility and plant growth, and it may increase their tolerance to stress, similarly as it was shown after the use of ash from other crops^9, 45–47^. The addition of APOL-HUMUS and Stymjod bio-preparations as well as the tested biogas plant digestate increased the beneficial effect of these fertilizers on plant growth and biomass yield. Research show that sorghum ash is slightly more effective in increasing sorghum plant development than ash from Jerusalem artichoke enriched with bio-preparations, which was studied in earlier work by Romanowska-Duda et al.^14^. The obtained results are in line with Biel et al.^47^ research who demonstrated the positive impact of ash from conifers on dry matter, crude protein and crude ash content in tubers of Jerusalem artichoke. An and Park^48^ showed that wood ash can be a soil amender counteracting the forest soil pH and a natural agent improving plant growth and element content in leaves. The additional use of nitrogen corrected nutrient soil deficiencies in soils, similarly as it was shown in the presented study where supplementation of sorghum ash with digestate enhanced more plant growth and soil fertility. The research of Bang-Andreasen et al.^10^ demonstrated also that wood ash strongly increased soil pH and electrical conductivity. Bacterial numbers strongly increased up to a wood ash dose of 22 t ha^−1^ followed by significant decrease at higher amounts. The quantity and species composition of bacteria significantly decreased with increasing amount of wood ash applied, probably due to ash induced changes in pH, electrical conductivity and the addition of nutrients to ash. These data are consistent with the authors’ preliminary results indicating that sorghum ash doses above 5 t ha^−1^ were less favorable and therefore 4 t ha^−1^ was tested in the presented study. Fertilization with ash as a way to increase plant growth was also indicated by Knapp and Insam^49^ and Wierzbowska et al.^45^. They concluded, that this dust contained several nutrients and of some of them it can be a source comparable to that of highly soluble commercial fertilizers. According to Stankowski et al.^50^ the ash from combusted plants, applied alone or with compost, increased ear density and yield of wheat, while Romanowska-Duda et al.^51^ reported that sorghum ash increased growth and physiological properties of macrophytes. However, the cited literature demonstrates the effect of ash from some particular plant species on some crops only. There is no strict indication of the possibility of replacing an artificial fertilization with ash from biomass in sorghum crops.

Application of natural ash from sorghum, used alone or together with the waste from corn grain biodigestion to methane, APOL-HUMUS and Stymjod, made it possible to reduce by half the recommended doses of artificial fertilizer YaraMila Complex and thus to limit the amount of chemical substances added to the soil. Replacing synthetic fertilizers with biomass ash decreases the environment pollution with harmful substances and enables ecological production of sorghum, free of toxic impurities. Based on the obtained results it can be assumed that natural sorghum ash can be used as a fertilizer in subsequent sorghum crops in a closed monocultural production of this species. The lack of toxic substances in plants, as an effect of fertilizing with ash, may also promote better absorption of various phytochemicals from sorghum by humans, although research in this field has not been conducted^52^. The results of Grzesik and Romanowska-Duda^53^ indicating the possibility of reducing chemical fertilization by the application of algae monocultures selected from inland water reservoirs to plants are further evidence supporting the possibility to exchange harmful fertilizers with natural compounds in plant production.

The demonstrated dependencies between the applied doses of sorghum ash and YaraMila Complex and plant growth were also confirmed by measurements of gas exchange activities which play a key role in sorghum development, as it was also shown by Abreha et al.^29^ and the scientists cited by them who studied the mechanisms of sorghum response to drought-stress involving morphological, physiological, and molecular alterations. The found dependence occurred in all cultivation conditions, as it was confirmed by 3-year research in the different soil quality and climate of Central and Northern Poland. The observed more intensive sorghum development in northern Poland than in its central part was probably caused by more favorable weather conditions and higher fertility of the podzolic soil then of sandy soil in all experimental variants, as it was also demonstrated after plant fertilization with ash from Jerusalem artichoke in similar conditions^14^.

The performed study shows that the nutrients contained in sorghum ash and YaraMila Complex had a positive effect on the photochemical processes of photosynthesis, which in turn stimulated plant growth. The found dependence is consistent with the research of Kalaji et al.^54^ indicating that the content of basic elements in corn and tomato plants determined the activity of the photosynthetic system. In the presented research, the potassium contained in the studied ash and YaraMila Complex could influence the activity of enzymes which take part in photosynthesis, regulation of assimilate transport and stomata opening. Phosphorus had a positive impact on the phosphorylation process, and iron on the photosynthesis intensity and it prevented leaf chlorosis. Magnesium, as being present in chlorophyll, the leaves greener. As shown in the presented research, the used ash from sorghum and YaraMila Complex contain significant amounts of macro and microelements that favorably affected photosynthetic processes, which resulted in the increased gas exchange and accelerated plant growth.

The sorghum ash and YaraMila Complex affected the chlorophyll content in leaves in proportion to the doses of these fertilizers. The content of chlorophyll could be an indicator of N content in sorghum leaves and their greenness, as it was observed by Schlemmer et al.^55^ assessing chlorophyll and nitrogen content in maize. This is in line with the presented research which showed that the increased content of chlorophyll in the leaves, after application of the studied sorghum ash and YaraMila Complex was linked with plant growth.

The studies showed that enhanced plant growth was associated with the increased activity of both phosphatases used for metabolic assessment, RNase and total dehydrogenases, correspondingly to the used doses of sorghum ash and YaraMila Complex. These correlations result from the role played by the assessed enzymes in regulating plant development. Phosphatases play important roles in plant signalling pathways, including pathogen defence and stress regulation, light and hormonal signalling, cell cycle and differentiation, metabolism, plant growth, phosphorus circulation in tissues and its mineralization^56^. The enzyme RNase takes part in strengthening of defense mechanisms in tissues, as it was observed in corn and willow treated with algae. Dehydrogenases play the important role in respiration processes^14^. The used sorghum ash enhanced the tested enzymatic activity, which resulted in more intense growth of plants in various environmental conditions. The similar dependence between the studied enzyme activities and plant growth was also demonstrated in corn and willow after their foliar spray with selected algae *Microcystis aeruginosa*, *Chlorella* sp. and *Anabaena* sp. collected from freshwater reservoirs in Poland, and in sorghum and Jerusalem artichoke fertilized with sewage sludge and digestate from biogas plant^14, 53^.

The studies show that the application of bio-fertilizers can result in better leaf quality and also makes them greener and healthier than in untreated plants. They also show that fertilization of plants with sorghum ash in the tested doses had no visible effect on the content of nutrients in sorghum leaves, except for potassium. On the other hand, the YaraMila application slightly increased the content of N, P and Mg in them. This is in line with the studies of Monti et al.^57^ who demonstrated a relatively high content of N in sorghum. They concluded also that the leaves were lower quality fuel than the stems and seed heads. Therefore, delaying the harvest of plants reduces the proportion of leaves in the biomass compared to the stems and improves the suitability of these biofuels for combustion. Zapałowska et al.^58^ showed that the applied coniferous tree ash had not significant influence on the content of Mg, Ca, C, N, P and S in shoots of Jerusalem artichoke, with the exception of K, whose amount increased by 30%. Wood ash also did not affect the content of the studied nutrients in willow plants, although after such fertilization the biomass yield was higher^59^. The presented research show that the elemental composition of sorghum ash mainly causes an increase in biomass yield and improves the development and health of plants, as was found after the use of Jerusalem artichoke ash together with biopreparations and digestate in sorghum^14^. The tested biological fertilizers, beside increasing the quality and greenery of plants, could also enhance their resistance to pathogen attack^37, 60^. They have an ability to boost the synthesis of components which can, e.g. increase the biosynthesis of lignin and suberin in cell walls. Lignin, cutin, and suberin contain a variety of organic compounds which strengthen cell walls and make them more resistant to fungal and bacterial attack by hindering the penetration of these pathogens into tissues and favouring the higher plant health^61^. This indicate the legitimacy of plant fertilization with the tested ash, which limits the need for use of chemical fertilizers and pesticides and has a positive effect on sorghum growth.

Fertilizing with sorghum ash slightly increases the energy value of plant biomass and limits ash content after their combustion compared to the control. This confirms the legitimacy of the use of this ash in sorghum crops for increasing the biomass yield, as it was found also by Szufa et al.^3^ who showed that fertilization with digestate enhanced the Jerusalem artichoke plant energy properties. The found dependence showing that slightly higher energy properties of plant biomass are inversely proportional to the ash content obtained from it is in line with the research of Demirbas^62^. He showed that the high ash content in plants reduced biomass energy parameters and made it less useful as fuel. In the presented study slight changes in energy parameters were found and thus sorghum fertilized with the studied sorghum ash can be cultivated for energy needs.

The performed research shows that the effectiveness of sorghum ash application, alone or in combination with various doses of synthetic YaraMila Complex, can be increased by supplementation with digestate from biogas plants and the used biopreparations, regardless of environmental conditions. Digestate from biogas plant increased the growth dynamics and yield of Jerusalem artichoke biomass cultivated in the closed circular system on poor soil not enriched with artificial fertilizers^3^. The beneficial effect of the soil improver APOL-HUMUS on plant growth could be due to the activity of chitosan polymers and humic acids, whose positive effect on plants is known. In the case of Stymjod, on the other hand, a positive impact on growth is exerted by its components: nutrients, humic acids and iodine. The stimulatory impact of humic acids on plant growth was shown by Man-hong et al.^63^ and the researchers cited by them. The beneficial effect of chitosan application on plant growth and health was widely described by Chakraborty et al.^64^ showing respective literature data known so far. Iodine, present in Stymjod, positively affected the cyto-morphological changes in plants. According to Jeznach^65^ research the iodine present in Stymjod increased xylem diameter in tomato and cabbage and caused the higher frequency of stomata opening and enhanced gas exchange in leaves. He reported that cabbage plants sprayed with iodine were more resistant to stress and they contained more macro-elements. This is in line with the review presented by Medrano-Macías et al.^66^ providing an overview of the literature data concerning the positive effect of iodine on plant growth and their stress tolerance. Contrary, Krzepiłko et al.^67^ demonstrated that iodine biofortification of lettuce reduced the seedling height, although it did not influence their biomass and the content of chlorophyll, calcium, copper, zinc and iron. However, they contained more potassium and less manganese and sodium than in control variant. Diverse impact of iodine on development of the mentioned plants could be caused by differences in doses, reaction of particular species and properties of the applied formulation which contain this element. The Stymjod bio-preparation was produced in modern specialist apparatus in which a mixture of mineral and organic compounds and iodine was subjected to cold synthesis^64^, while in other cited studies iodine was used alone or in the form of simple chemical compounds. The clarification of differences in iodine activities presented in this research requires further studies in this field, including production methods of preparations containing this element.

**Figure 4:**
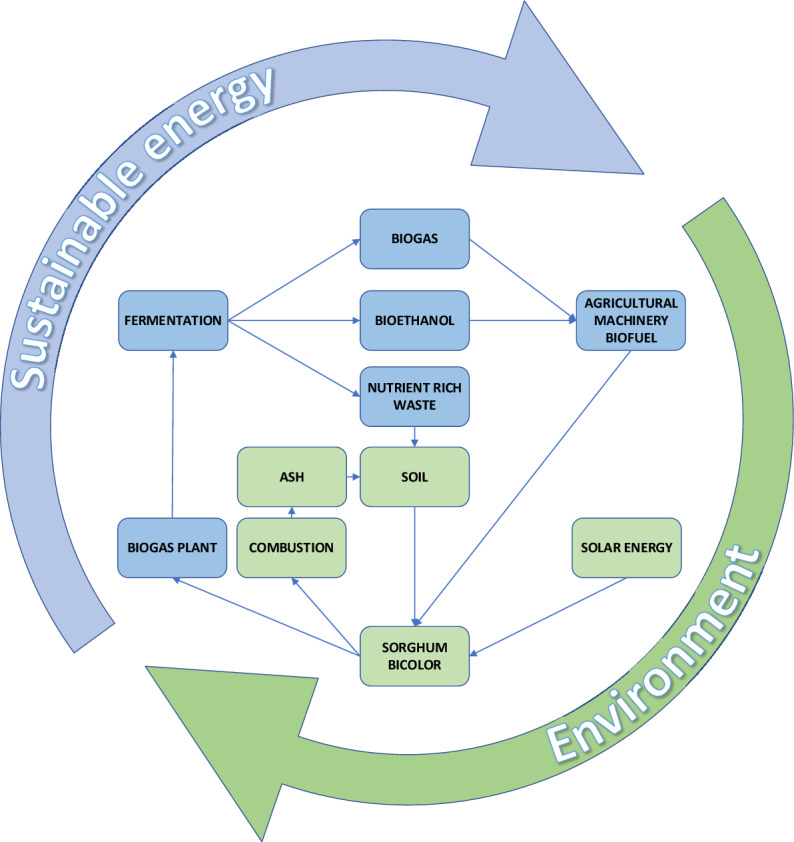
The circulating cycle of nutrient dislocation from sorghum biomass to waste after its digestion to methane or to ash after combustion, then to soil and back to plants in a closed crop production system.

The studies show that natural sorghum ash can be used as a fertilizer of energy crops in different soil quality and climate of central and northern Poland. Its use allows to limit the recommended doses of chemical fertilizers hazardous for environment. A similar effect was obtained after the use of Jerusalem artichoke ash, which, however, had a slightly less favorable effect on development and physiological activity than sorghum ash tested in the presented studies^14^. The results of the present research also indicate the possibility of producing sorghum in a closed cycle, in which waste becomes raw material in the next rotation, as suggested also by Knapp and Insam^49^ discussing various strategies and technologies for recycling waste, including biomass ash, from various sources. In this production the obtained biomass can be combusted to obtain thermal energy and the obtained ash may be used to fertilize the crops in the following year. This biomass can be also used as a raw material for biogas and biofuel production and the resulting digestate to fertilize the new plants in the next cultivation cycle on the same area. Thus, in both cases a closed circulation of nutrients take place, maintaining high fertility of the soil and allowing to limit the use of harmful chemical fertilization and pesticides. Thanks to the presented research, the possibility of using sorghum ash as a fertilizer in subsequent sorghum crops was proved. This makes long-term, closed, environmentally friendly monoculture production of this energy plant possible, due to the reduction of chemical fertilizers while maintaining high soil fertility and high yields of biomass (Fig. 4). This findings become important because amounts of biomass ash are rapidly growing and thus their ecological and economical use in agriculture becomes to be one of the most rational goals. It is particularly important in the cultivation of energy crops, where biomass ash, even containing heavy metals, can be used with sewage sludge as fertilizer. This enable waste management reducing the loss of natural resources which has become a basis of sustainable development and a circular economy, as indicated by Antonkiewicz et al.^68^. According to these authors the prepared waste mixtures containing coal or biomass ash and municipal sewage sludge would reduce the environmental risk compared to the studied waste used separately.

## Conclusion

One of the most important needs of modern agriculture is to limit artificial fertilization by using natural fertilizers that do not contain toxic compounds and do not pollute the environment and plants. The presented research helps to achieve these goals and shows that the use of ash from combusted plant biomass for fertilization purposes is an excellent solution to the problem of storing this waste and ensures a closed circulation of nutrients between the plant and the soil, increasing its fertility. The obtained results indicate the possibility of ecological and economical fertilization of energy sorghum crops with natural ash from sorghum biomass, used alone or supplemented with digestate from the biofermentation of maize grain to methane, APOL-HUMUS as a soil improver and Stymjod, a nano-organic-mineral fertilizer, in order to reduce/eliminate the use of synthetic fertilizers in a closed system of biomass production of sorghum plants for energy purposes.

## Data Availability

The manuscript provides averages from many experiments and years, which accurately demonstrate the effect of the developed ash fertilization, applied separately or together with digestate and biopreparations, on the development and physiological activity of sorghum. The presented results give grounds for their use in practice, provided that they are adapted to specific cultivation conditions and show the important information for science in the field of plant response to the applied treatments. However, the detailed results will be used by the authors for further studies, which will constitute the content of the patent and at the same time can be used directly in the companies where the research was conducted. For commercial reasons, these companies and other contractors do not agree to share detailed results that would enable their direct use in similar cultivation conditions. Prof. Mieczysław Grzesik, The National Institute of Horticultural Research in Poland; Mieczyslaw.Grzesik@inhort.pl can be contacted.
